# Supplementary material for: Expectation violations as an effective alternative to complex mentalizing in novel communication
Source: iScience. 2025 May 30;28(7):112795. doi: 10.1016/j.isci.2025.112795 (PMC12209988; doi:10.1016/j.isci.2025.112795)
Supplement: Document S1. Figures S1 and S2 [file mmc1.pdf]

iScience, Volume *28*

Supplemental information

Expectation violations as an effective alternative to complex mentalizing in novel communication

Tatia Buidze, Yuan-Wei Yao, and Jan Gläscher

Supplement figures:

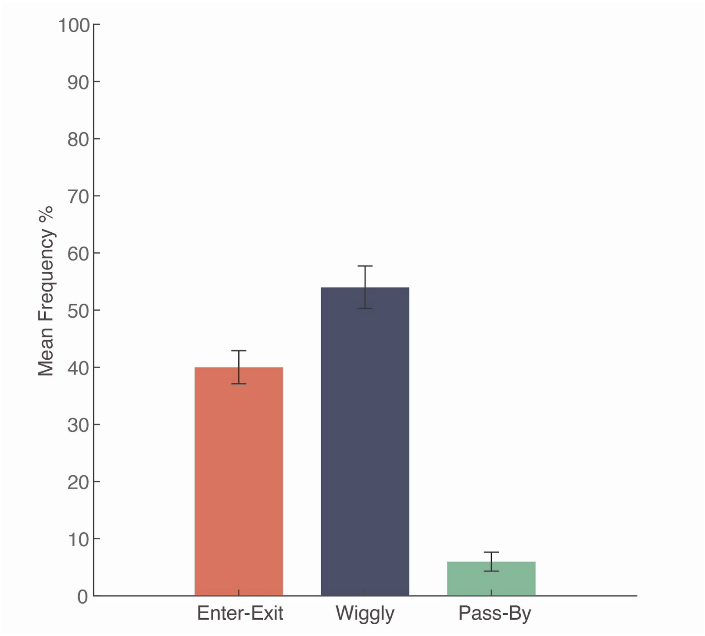

Figure S1. Individually fitted Surprise-model parameters reproduce human Pass-By messages (related to Figure 3). The Surprise model was re-simulated with parameters fitted separately for each of the 40 human Senders (rather than the group-average parameters used in the main text). Bars show the mean percentage of Enter-Exit, Wiggly, and Pass-By trajectories generated under these individual fits; error bars indicate ± SEM across participants.

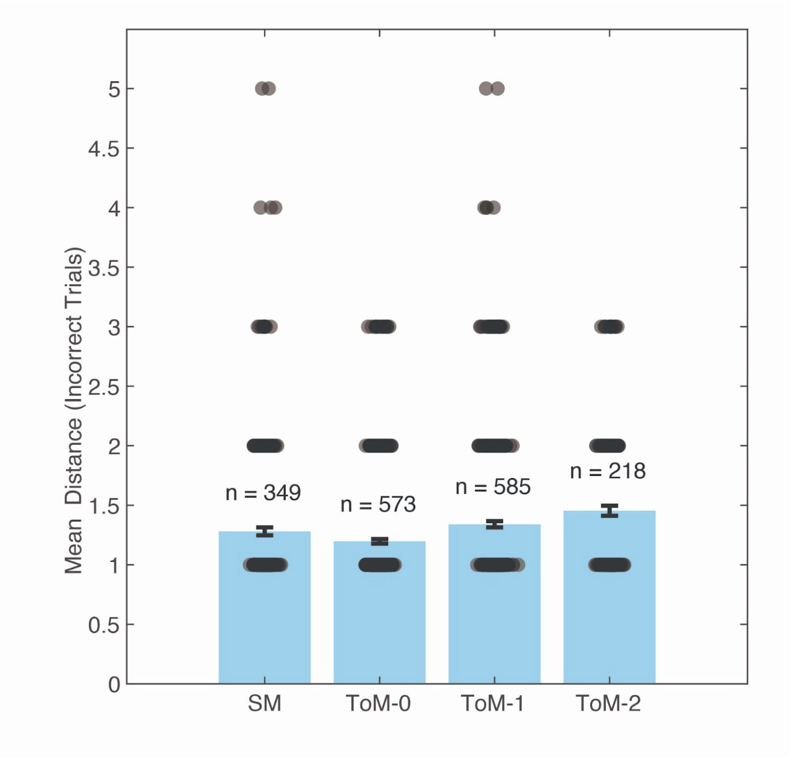

Figure S2. Receiver error-distance analysis across sender models (related to Figure 4). For each incorrect trial we computed the grid-distance between the Receiver's chosen tile and the true goal. Bars depict the mean error distance for each model (Surprise, ToM-0, ToM-1, ToM-2); dots represent individual trials. Error distances were broadly comparable across models, although ToM-2 shows a slightly higher average because its highly effective Enter-Exit messages produced very few errors, when errors did occur, they reflected early-phase misunderstandings rather than near-misses. In contrast, the Surprise, ToM-0 and ToM-1 models generated more ambiguous Wiggly or Pass-By messages, resulting in more frequent but typically closer errors.
